# Application of mesohepatectomy with caudate lobectomy for the treatment of type III–IV hilar cholangiocarcinoma: a single-center retrospective study

**DOI:** 10.1186/s40001-023-01209-0

**Published:** 2023-07-13

**Authors:** Dongdong Wang, Wanliang Sun, Shuo Zhou, Zhong Liu, Zheng Lu, Dengyong Zhang

**Affiliations:** Department of General Surgery, The First Affiliated Hospital of Bengbu Medical College, No.287 Chang Huai Road, Bengbu, 233000 Anhui China

**Keywords:** Hilar cholangiocarcinoma, Mesohepatectomy, Hemihepatectomy, Survival

## Abstract

**Background:**

The main surgical procedure for Bismuth‒Corlette III–IV hilar cholangiocarcinoma (HCCA) is hemihepatectomy/extended hemihepatectomy. However, many patients have no opportunity for surgery due to having an insufficient remnant liver volume. Preservation of more liver volume on the premise of ensuring R0 resection is the goal. Mesohepatectomy with caudate lobectomy may be a new method to meet these requirements.

**Methods:**

The clinical data of 41 patients with Bismuth‒Corlette III–IV HCCA, including 18 patients who underwent mesohepatectomy with caudate lobectomy (the mesohepatectomy group) and 23 patients who underwent hemihepatectomy or extended hemihepatectomy (the hemihepatectomy group), were analyzed retrospectively. The perioperative indicators and prognostic survival time between the two groups were analyzed.

**Results:**

The mesohepatectomy group was compared with the hemihepatectomy group, and the operation time was 7.95 ± 1.2 vs. 7.15 ± 1.5 h (*P* > 0.05); the intraoperative blood loss was 600.0 ± 153.4 vs. 846.1 ± 366.8 mL (*P* < 0.05); the postoperative hospital stay was 9.9 ± 2.2 vs. 13.8 ± 3.0 days (*P* < 0.05); and the R0 resection rate was 100% vs. 87.0% (*P* > 0.05). The postoperative complications of the two groups included bile leakage (22.2% vs. 21.7%), pleural effusion (11.1% vs. 8.7%), and fever (16.7% vs. 8.7%), with no significant differences in the incidences (*P* > 0.05). The 1-, 3-, and 5-year survival rates of the two groups were 87.5%, 55.7%, 27.8% and 83.5%, 56.1%, 24.5%, respectively, with no significant differences (*P* > 0.05).

**Conclusions:**

Mesohepatectomy with caudate lobectomy can preserve more functional liver volume while ensuring the bile duct margin. It can be applied as the surgical treatment of Bismuth‒Corlette III–IV HCCA.

## Introduction

Hilar cholangiocarcinoma (HCCA) is a malignant tumor that is located in the bile duct epithelium, extending from the left and right hepatic ducts to the opening of the cystic duct, accounting for 50–70% of all cholangiocarcinomas [1]. Bismuth and Corlette divided HCCA into four types according to the location of the lesion. In type I, the tumor is located in the common hepatic duct and does not invade the confluence of the left and right hepatic ducts; in type II, the tumor invades the confluence and does not invade the left or right hepatic duct; in type III a, the tumor invades the right hepatic duct; in type III b, the tumor invades the left hepatic duct; and in type IV, the tumor invades both the left and right hepatic ducts [2]. Large-scale hepatectomy, such as hemihepatectomy, left trilobal resection or right trilobal resection, is always used for HCCA, but these operations involve a large volume of liver resection and may result in a high incidence of complications, such as liver failure and death [3]. Another operation is perihilar resection [4], which is suitable for short-distance bile duct invasion, such as Bismuth‒Corlette type II HCCA. Moreover, the extent of liver resection in perihilar resection is not fixed, and there is no unified or defined extent of resection in hepatectomy. As a result of perihilar resection, there are many broken ends of the bile duct and many biliary-intestinal anastomoses, which are difficult to eradicate during the operation and are prone to bile leakage complications after the operation. With the development of the concept of precision surgery and the progress of modern science and technology, HCCA is progressing toward becoming a precision surgery, and the extent of HCCA invasion, the boundary of bile duct resection and the accurate measurement of the liver volume can be accurately evaluated before the operation. This provides a reference for guiding individualized surgical methods. We found a new operation for HCCA and have discussed it below.

In this study, we enrolled patients with Bismuth‒Corlette type III–IV HCCAs that invaded the common hepatic duct, the confluence of the left and right hepatic ducts, and the left and/or right hepatic ducts but did not invade the secondary bile duct branches. At present, the most effective method for the treatment of HCCAs is surgical R0 resection, which involves resection of the diseased bile ducts, nearby liver tissues, nerves, and lymphoid tissues [5]. Currently, surgeons believe that the best surgical method for treating Bismuth‒Corlette type III–IV HCCAs is mainly hemihepatectomy/extended hemihepatectomy with caudate lobectomy [6]. However, to avoid liver failure after the resection of a large volume of the liver, patients need a longer period of jaundice treatment before surgery. If the volume of the remnant functional liver is insufficient, staged surgery such as ALPPS (associating liver partition and portal vein ligation for staged hepatectomy), portal vein embolization or hepatic venous deprivation can be performed to help increase the volume of the remnant liver. Mesohepatectomy was first proposed by McBride in 1972. The middle liver lobe includes Couinaud classified segments 4a, 4b, 5, and 8 [7]. At present, mesohepatectomy has been used for the treatment of liver cancer, but its application for the treatment of HCCAs remains to be explored. In this study, we reported the clinical experience at a single center by comparing and analyzing the efficacies of mesohepatectomy and extended hemihepatectomy/hemihepatectomy in the treatment of Bismuth‒Corlette type III–IV HCCA.

## Patients and methods

### Inclusion criteria and exclusion criteria

The clinical data of 41 patients with Bismuth‒Corlette type III–IV HCCAs who underwent surgical treatment at the General Surgery Department, the First Affiliated Hospital of Bengbu Medical College, from January 2012 to July 2022 were collected. Inclusion criteria were as follows: patients with Bismuth‒Corlette type III–IV HCCAs that were diagnosed through 3D CT and other preoperative clinical symptoms; patients who underwent surgical treatment; patients with a pathological diagnosis of adenocarcinoma postoperatively; patients with a tumor that shows no distant metastasis; and patients with no other surgical contraindications. The exclusion criteria were as follows: patients with other organ metastases; patients who could not tolerate the surgery; patients who could not cooperate with the treatment; and patients with missing follow-up or incomplete information. The study protocol was approved by the Ethics Committee of Bengbu Medical College.

### Preoperative evaluation

**Fig. 1:**
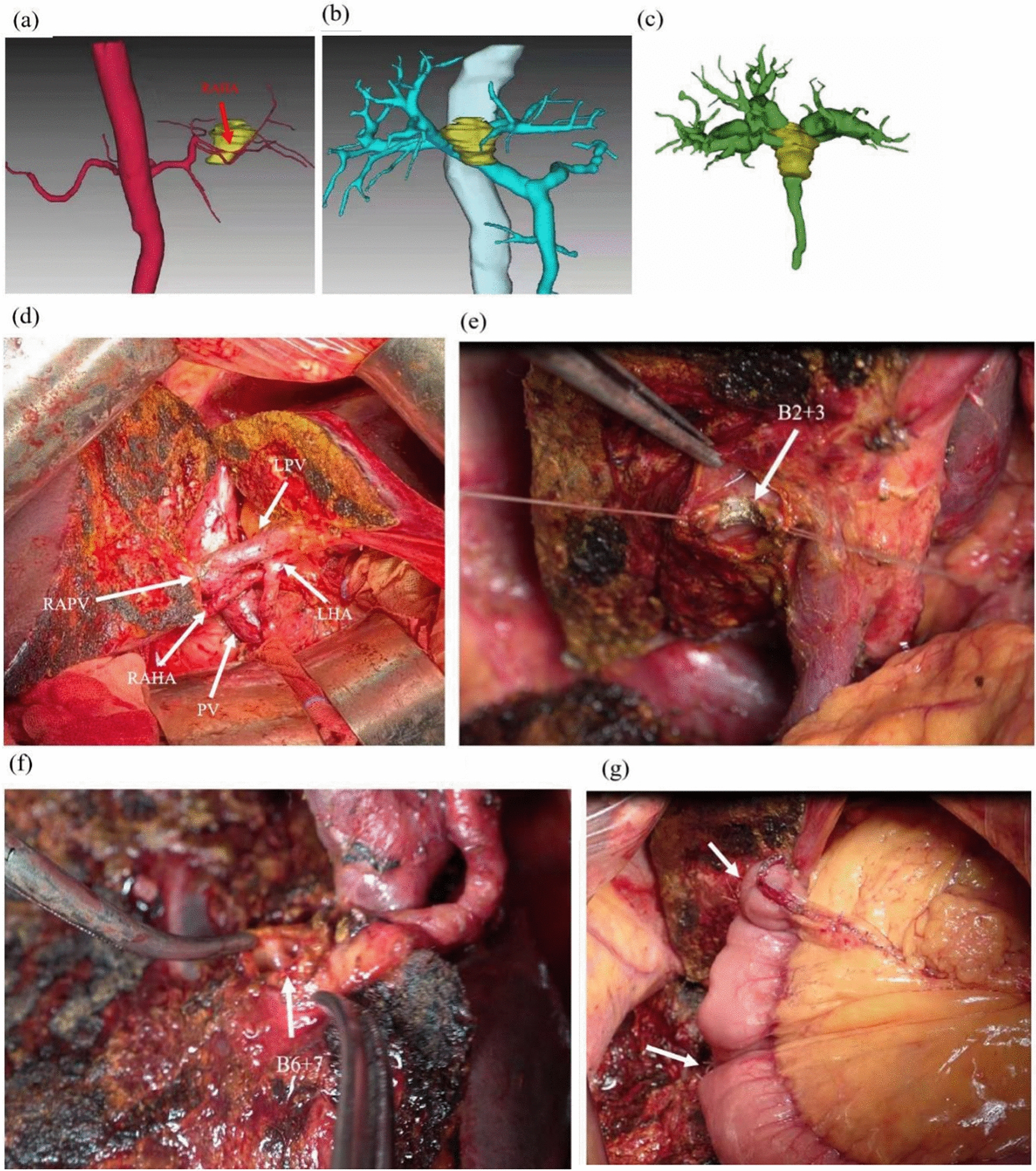
Imaging data of one patient who underwent mesohepatectomy with caudate lobectomy. The 3D reconstruction model showed that the tumor (yellow) invaded the right anterior branch of the hepatic artery (**a**). The tumor clung to the roots of the left portal vein and right anterior portal vein (**b**). The location of the tumors in relation to the bile duct (**c**). Anatomical structure after mesohepatectomy with caudate lobectomy (**d**). Shaping the B2 and B3 biliary branches, B6 and B7 biliary branches, respectively (**e**–**f**). Two choledochojejunostomy operations were performed (white arrow) (**g**). RAHA: right anterior branch hepatic artery; *PV* portal vein, *LPV* left hepatic portal vein, *RAPV* right anterior branch hepatic portal vein, *LHA* left hepatic artery

All patients underwent a thin-slice CT scan before the operation to obtain image data. The CT data were imported into 3D visualization imaging software to complete cutting and reconstruction to obtain the 3D model (Fig. 1A–C). According to the 3D model, the HCCAs can be accurately classified; preoperative surgical planning can also be performed to clarify the anatomy of the intrahepatic vessels, tumors and the whole liver and to determine whether there were anatomical variations; the volume of each liver segment was accurately calculated before the operation, and the remnant liver volume was accurately calculated. Patients who met the general conditions described in Mansour’s paper were enrolled in the hemihepatectomy group [8]. Patients who met other conditions including, no tumor invasion in the right posterior branch, left external branch of the portal vein, right hepatic artery, right posterior hepatic artery, left hepatic artery or left external hepatic artery.

### Surgical methods

#### Mesohepatectomy with caudate lobectomy

The skin was incised to open the abdominal cavity using a reverse L-shaped incision in the upper abdomen. First, the location of the tumor was explored to confirm that there was no tumor metastasis in the abdominal cavity. The preperitoneum of the hepatoduodenal ligament was incised at the upper edge of the duodenum, the proper hepatic artery was dissected and separated, and the hepatic artery was suspended with a thin rubber tube, which was used to separate downward along the hepatic artery to the confluence of the gastroduodenal artery (GDA). The right hepatic artery, right posterior hepatic artery, left hepatic artery, and left lateral hepatic artery were separated upward along the hepatic artery to confirm that these vessels were not invaded by the tumor. The common bile duct was ligated and divided, the bile duct was retracted upward, and then the lymph, nerve, and adipose tissue inside the hepatic artery were dissected. The portal venous trunk, left portal vein, extrahepatic left portal vein, right portal vein, and right posterior hepatic portal vein were dissected and separated to confirm that these vessels were not invaded by the tumor. The roots of the right anterior hepatic artery, left hepatic artery, right anterior hepatic portal vein and left hepatic portal vein were all ligated and then divided. At this time, a demarcation line can be seen on the liver surface. The liver was fully mobilized, and several short hepatic veins were cut off. The vessels supplying the caudate lobe from the portal venous trunk were ligated and divided, and the caudate lobe was completely separated from the first porta hepatis.

For resection, the gallbladder was removed first, and then the resection line was drawn. Resection was performed along the boundary of the middle liver lobe and the marked line. The branches of segments II and III (B2 + 3) of the left hepatic bile duct and the right posterior bile duct (B6 + 7) of the right liver were preserved. The specimen was completely resected together with the caudate lobe (clear anatomical structure can be seen after resection, Fig. 1D). The jejunum was transected approximately 15 cm below the ligament of Treitz. The distal jejunum was sutured and closed and then lifted to the first porta hepatis through the colon. Approximately 5 cm from the blind end of the jejunum, 1.0 cm of the jejunum was cut to allow end-to-side anastomosis with the left hepatic bile duct branch (two bile ducts were sutured and reshaped into one bile duct before anastomosis, B2 + 3, Fig. 1E). Choledochojejunostomy was performed with the right posterior bile duct at an appropriate length of the jejunum below the first biliary-intestinal anastomotic stoma (B6 + 7, Fig. 1F). Two choledochojejunostomy operations were performed (Fig. 1G). Finally, 3.0 cm of the jejunum was cut approximately 50 cm below the second anastomotic stoma to allow end-to-side anastomosis with the proximal broken end of the jejunum.

#### Hemihepatectomy/extended hemihepatectomy

The mobilization and dissection procedures in hemihepatectomy/extended hemihepatectomy with caudate lobectomy were the same as those in mesohepatectomy with caudate lobectomy. During resection, only liver segments 1 + 4 + 5 + 6 + 7 + 8, segments 1 + 2 + 3 + 4 + 5 + 8, segments 1 + 5 + 6 + 7 + 8 or segments 1 + 2 + 3 + 4 were resected.

#### Data collection and statistical analysis

The general data of the two groups of patients were collected and compared, such as preoperative indicators, intraoperative blood loss, operation time, postoperative complications, hospital stay, R0 resection rate, and 1-, 3-, and 5-year survival rates. SPSS 25.0 software was used to analyze the collected data, and the measurement data between groups were first analyzed by Levene’s test of homogeneity of variance (the F test). If the two sets of data were normally distributed, the t test was used; otherwise, the approximate t test was used. The survival times of the patients were recorded, and the Kaplan‒Meier survival curve was plotted. A *P* value < 0.05 was considered statistically significant.

## Results

### General data

There were 18 patients in the mesohepatectomy group, including 11 males and 7 females, with an average age of 53.4 ± 9.8 years; there were 23 patients in the hemihepatectomy group, including 14 males and 9 females, with an average age of 57.7 ± 11.7 years. Liver function was evaluated using Child‒Pugh grading. In the mesohepatectomy group, 6 patients had grade A liver function and 12 patients had grade B; in the hemihepatectomy group, 8 patients had grade A liver function and 15 patients with grade B. There was no significant difference in age, sex, liver function (Child‒Pugh), preoperative bilirubin, planned resection liver volume, hepatitis, preoperative ALT, preoperative AST, preoperative ALP, CA199, or percutaneous transhepatic biliary drainage (PTBD) between the patients who underwent the two surgical methods (all *P* > 0.05, Table 1). None of the patients had cirrhosis in either group.

**Table 1:** General clinical data of the patients before the operation

Indicator	Mesohepatectomy group (*n* = 18)	Hemihepatectomy group (* n* = 23)	P value
Age (years)	53.4 ± 9.8	57.7 ± 11.7	0.216
Sex (male/female)	11/7	14/9	0.987
Child‒Pugh (A/B)	6/12	8/15	0.923
Preoperative liver function			
Child‒Pugh A	6	8	
Child‒Pugh B	12	15	
Bismuth‒Corlette grading11/7	11/12	0.397	
III (A/B)	11(1/10)	11(5/6)	
IV	7	12	
Preoperative bilirubin	83.5 ± 16.1	75.2 ± 12.0	0.065
Planned resection liver volume	864.6 ± 119.0	1136.7 ± 243.4	0
Hepatitis (Y/N)	2/16	3/20	1.000
Preoperative ALT	101.4 ± 35.7	98.3 ± 53.5	0.831
Preoperative AST	89.2 ± 21.7	86.1 ± 42.4	0.761
Preoperative ALP	212.8 ± 44.5	200.5 ± 34.1	0.322
CA199	450.3 ± 276.7	400.1 ± 265.4	0.559
PTBD (Y/N)	15/3	21/2	0.769

### Perioperative indicators

Forty-one cases were confirmed to be Bismuth‒Corlette type III–IV HCCAs by preoperative imaging and intraoperative examination. The operation times of mesohepatectomy with caudate lobectomy and hemihepatectomy/extended hemihepatectomy with caudate lobectomy were 7.95 ± 1.2. 7.15 ± 1.5 h, respectively (*P* > 0.05); the intraoperative blood losses were 600.0 ± 153.4 mL and 846.1 ± 366.8 mL, respectively (*P* < 0.05); the postoperative hospital stays were 9.9 ± 2.2 d and 13.8 ± 3.0 d, respectively (*P* < 0.05); and the R0 resection rates were 100% (18/18) and 87.0% (20/23), respectively (*P* > 0.05). The pathological diagnosis of all patients in both groups was adenocarcinoma. The first hepatic portal occlusion time and postoperative lymph node count were not significantly different (Table 2). We performed first hepatic portal occlusion during liver resection, blocking for 15 min at once and then in 5 min intervals. Postoperative complications in the mesohepatectomy group included bile leakage (4 cases; 22.2%), pleural effusion (2 cases; 11.1%), and fever (3 cases; 16.7%). Postoperative complications in the hemihepatectomy group included bile leakage (5 cases; 21.7%), pleural effusion (2 cases; 8.7%), and fever (2 cases; 8.7%). None of the patients in either group had perioperative liver failure or died (Table 2).

**Table 2:** Comparison of data between mesohepatectomy with caudate lobectomy and hemihepatectomy/extended hemihepatectomy with caudate lobectomy

Indicator	Mesohepatectomy with caudate lobectomy (*n* = 18)	Hemihepatectomy/extended hemihepatectomy with caudate lobectomy (*n* = 23)	P value
Intraoperative blood loss (ml)	600.0 ± 153.4	846.1 ± 366.8	0.007
R0/R1 resection	18/0	20/3	0.323
Postoperative hospital stay (days)	9.9 ± 2.2	13.8 ± 3.0	0.000
Lymph node ( ±)	0/18	1/22	1.000
First hepatic portal occlusion	77.78 ± 11.01	75.38 ± 15.55	0.577

Time (minutes)

### Follow-up of patients

**Fig. 2:**
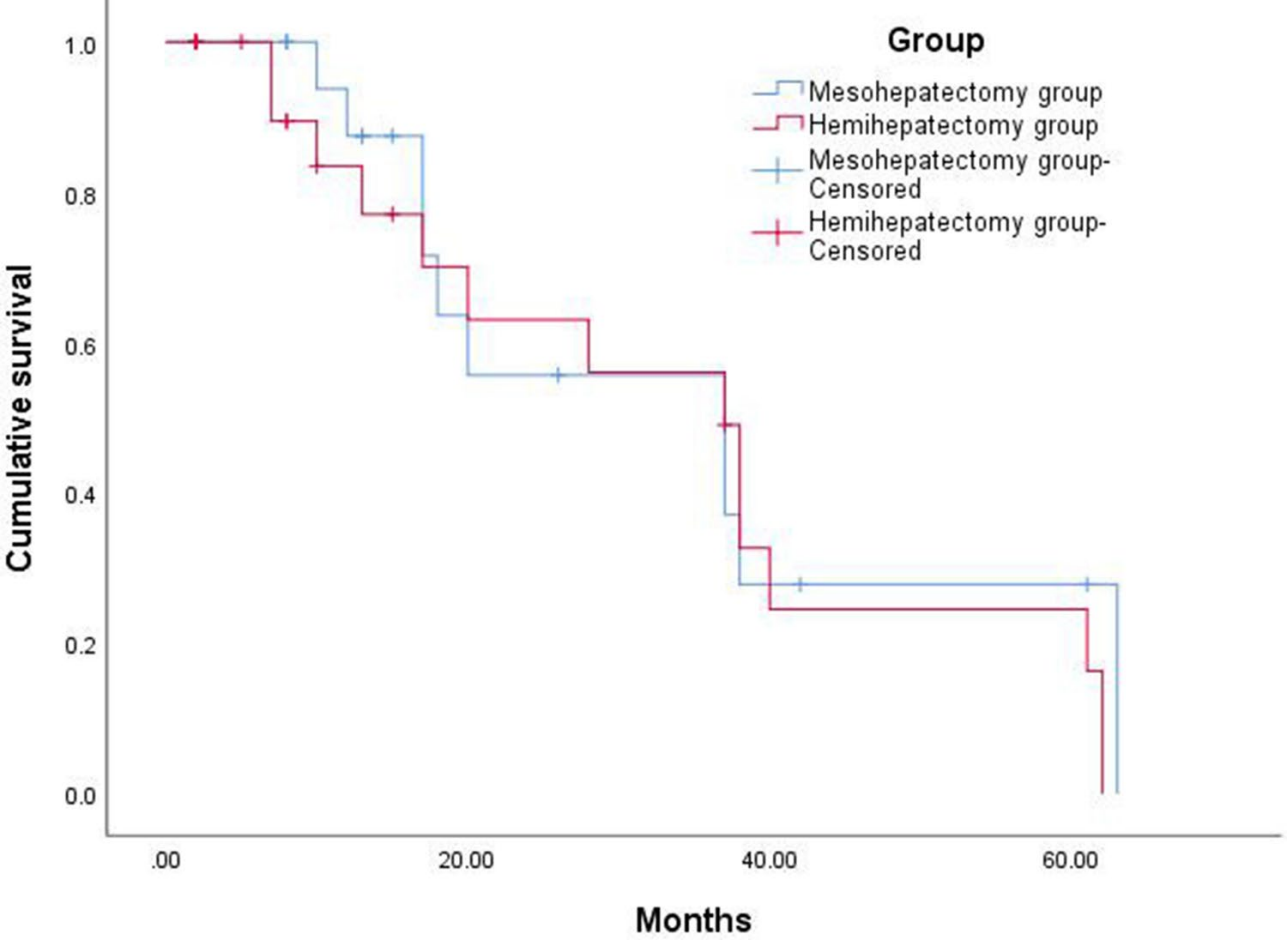
Overall survival curves of the patients in the two groups

The patients were followed up in either the in- or outpatient clinic or via telephone. A total of 43 patients were followed up, and 2 patients were lost to follow-up, with a total follow-up rate of 95.3%. Follow-up began in the first month after the operation. The patients’ liver functions and tumor indicators and the presence of tumor recurrence and metastasis were evaluated under postoperative imaging. The follow-up time was 1–63 months, with a median follow-up time of 37 months in both groups. Using Kaplan‒Meier survival function analysis, the 1-, 3-, and 5-year survival rates after mesohepatectomy with caudate lobectomy and hemihepatectomy/extended hemihepatectomy with caudate lobectomy were 87.5%, 55.7%, 27.8%, and 83.5%, 56.1%, 24.5%, respectively, and the difference was not statistically significant (Fig. 2).

## Discussion

At present, radical surgical resection is the main treatment for HCCAs [9]. Cholangiocarcinomas are known for longitudinal invasion of the bile duct and vertical invasion of nearby liver tissue and blood vessels. Hemihepatectomy/extended hemihepatectomy are effective standard operations for achieving negative bile duct margins [10]. For Bismuth‒Corlette type III–IV HCCAs, it has been widely recognized that combined caudate lobectomy can improve the radical resection rate and the postoperative survival time [11]. However, large-scale hepatectomy for the treatment of Bismuth‒Corlette type III–IV HCCAs can easily lead to an insufficient remnant liver volume, thus resulting in serious complications, such as postoperative liver failure. For patients with severe preoperative jaundice or an insufficient volume of the remnant functional liver based on preoperative assessment, preparations are needed; for example, preoperative ultrasound-guided PTBD is performed to relieve jaundice, and portal vein embolization therapy or ALPPS surgery is performed before the operation to preserve liver function. Delayed treatment may cause tumor progression, and the opportunity for surgical resection is lost. Some scholars have also used perihilar resection to treat HCCAs and preserve more liver volume [12]. However, this is a nonanatomical hepatectomy, and resection of the caudate lobe is difficult. The probability of postoperative biliary leakage is high, and the clinical prognosis is poor. Therefore, this surgical method has not been widely recognized.

**Fig. 3:**
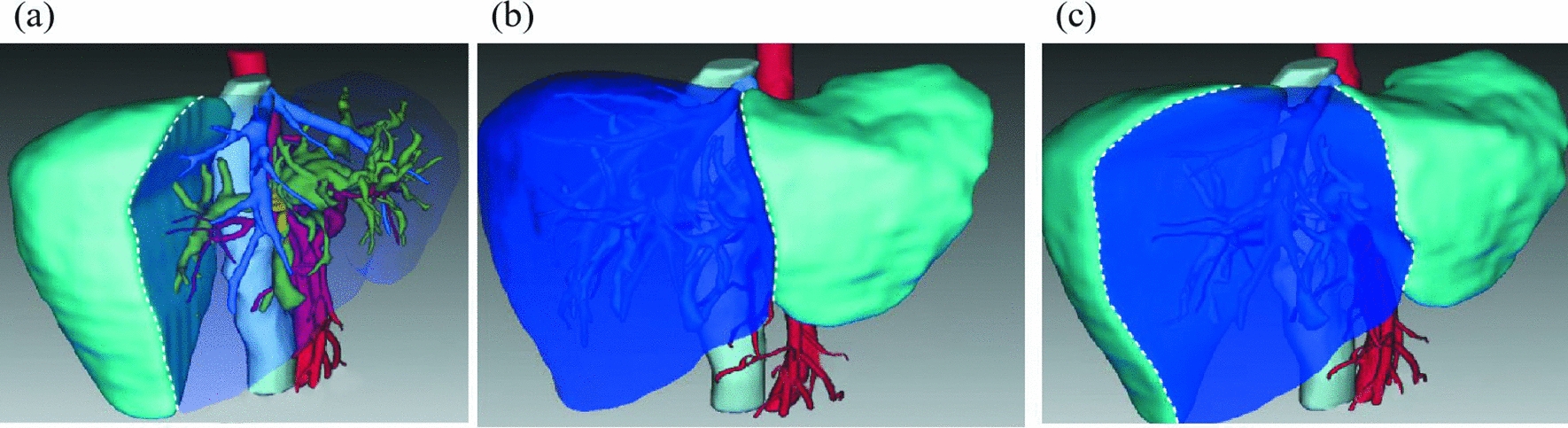
Three-dimensional reconstructive images showing the extent of hepatectomy with different approaches. **a** Extended left hemihepatectomy (Segments 1 + 2 + 3 + 4 + 5 + 8); **b** extended right hemihepatectomy (Segments 4 + 5 + 6 + 7 + 8); **c** bile duct incision edge of mesohepatectomy (Segments 1 + 4 + 5 + 8 resection). The bile duct incision edge of mesohepatectomy = the bile duct incision edge of enlarged left hemihepatectomy + enlarged right hemihepatectomy

In 1972, McBride and Wallace first described the resection of liver segments S4a, S4b, S5 and S8 for the treatment of gallbladder cancer [7]. In 1999, Wu et al. named this surgical resection method “mesohepatectomy” [13]. It is generally believed that the volume of the remnant functional liver after liver resection should be at least 30%. For patients with obstructive jaundice, the remnant functional liver volume should preferably be greater than 40% to ensure postoperative safety [14]. Studies have shown that the volume of the remnant functional liver was 25–30% after left hepatic trisectionectomy + caudate lobectomy, 15–20% after right hepatic trisectionectomy + caudate lobectomy and 45–50% after mesohepatectomy + caudate lobectomy [15]. These data suggest that more remnant functional liver volume can be preserved after mesohepatectomy with caudate lobectomy. Moreover, studies have shown that the incidence of liver failure after mesohepatectomy was significantly lower than that after right hepatic trisectionectomy [16]. In this study, for Bismuth‒Corlette type III–IV HCCAs, we found no significant difference in the postoperative overall survival rate between mesohepatectomy with caudate lobectomy and hemihepatectomy/extended hemihepatectomy with caudate lobectomy. In the mesohepatectomy group, intraoperative blood loss was significantly reduced, the postoperative hospital stay was significantly shortened, and radical resection was successful in all the patients. Figure 3 shows that mesohepatectomy allowed an extended the incisal edge of the bile duct when compared with left and right hemihepatectomy and ensured a pathologically negative resection margin after the operation. Our study suggests that mesohepatectomy with caudate lobectomy can preserve more remnant functional liver volume while ensuring the R0 resection rate, with less trauma and rapid postoperative recovery.

Studies have shown that a HCCA can invade 0.6–20.0 mm of the submucosa [17]. Based on the anatomy of the liver, the left and right hepatic ducts mainly converge on the right side of the porta hepatis, the left hepatic duct is relatively long, and the distance between the incisal edge of the U point at the branch of the left hepatic duct and the confluence of the left and right hepatic ducts is 1.6 cm. Therefore, the bile duct of the left hepatic lateral lobe is not easily invaded by the tumor, which provides a feasible basis for 4-segment resection in mesohepatectomy. Another study proved that the range of bile ducts in mesohepatectomy was significantly larger than that in hemihepatectomy [18]. This indicates that the R0 resection rate of mesohepatectomy is higher.

For patients with HCCAs, it is necessary to relieve jaundice before surgery to avoid the occurrence of postoperative complications. Some studies have shown that preoperative jaundice relief is required for patients with total bilirubin greater than 100 μmol/L, cholangitis, severe malnutrition, and a large scope of liver resection, which generally takes 2–3 weeks [19, 20]. In this study, we found that patients with Bismuth‒Corlette type III–IV HCCAs could undergo mesohepatectomy with caudate lobectomy when the preoperative total bilirubin did not exceed 150 μmol/L and there was no cholangitis or malnutrition. However, if such patients undergo extended hepatectomy, more than 50% of the remnant liver volume cannot be guaranteed, so a longer waiting time is needed, which increases the risk of tumor progression.

For patients with Bismuth‒Corlette type III–IV HCCAs, the tumor easily invades blood vessels [21]. Studies have shown that resection and reconstruction of the invaded portal vein in HCCA patients do not increase the risk of mortality but rather prolongs the patients’ postoperative survival times [22, 23]. Furthermore, studies have shown that resection and reconstruction of the invaded hepatic artery had no significant effect on the incidence of complications or the perioperative mortality of patients [24, 25]. The inclusion criteria of this study were as follows: patients with no invasion of the right posterior branch or the left external branch of the portal vein, and patients with no invasion of the right hepatic artery, right posterior hepatic artery, left hepatic artery or left external hepatic artery. The criteria have a high requirement for the hepatic artery. Mesohepatectomy with caudate lobectomy is not recommended when the tumor invades the right hepatic artery, right posterior hepatic artery, left hepatic artery or left external hepatic artery. In this study, in the mesohepatectomy group, the middle hepatic artery was invaded in 2 cases, and middle hepatic artery resection was performed during the operation. The portal venous trunk was invaded in 1 case, the right branch of the portal vein was invaded in 1 case, and both of them underwent partial resection and suture of the portal vein to achieve radical resection.

This study has several limitations. First, the number of cases in our study was small, especially in the mesohepatectomy with caudate lobectomy group, but hilar cholangiocarcinoma is rare, and we are accumulating cases and hope to publish this study in the near future. Second, we did not perform a statistical analysis according to tumor type (Bismuth‒Corlette type III–IV) due to the small number of cases. Third, perhaps it is inappropriate for us to compare the clinical data of two groups of patients, because there are some indicators that are different between the two groups, such as the right hepatic artery not being involved in all the cases in the mesohepatectomy group. Therefore, we need to continue to collect more cases to make the study more statistically significant.

## Conclusion

Mesohepatectomy with caudate lobectomy is advantageous for the treatment of Bismuth‒Corlette type III–IV HCCAs in that it can ensure a sufficient incisal edge of the bile duct to improve the R0 resection rate (100%), it can maximize the preservation of remnant functional liver volume and the requirement for preoperative total bilirubin level is not high, which reduces the preoperative preparation time and reduces the risk of tumor progression. Furthermore, the postoperative recovery time of patients is short, and the perioperative safety is high. We suggest that this surgical method can be applied in centers with experience in the surgical treatment of HCCAs.

## Data Availability

The data generated and/or analyzed during this study are available from the corresponding author on reasonable request.
